# Supplementary material for: The C. elegans neural editome reveals an ADAR target mRNA required for proper chemotaxis
Source: eLife. 2017 Sep 19;6:e28625. doi: 10.7554/eLife.28625 (PMC5644944; doi:10.7554/eLife.28625)
Supplement: Transparent reporting form [file elife-28625-transrepform.pdf]

1st Floor
24 Hills Road
Cambridge CB2 1JP, UK

P 01223 855340
W elifesciences.org
T @elife

eLife's transparent reporting form

We encourage authors to provide detailed information *within their submission* to facilitate the interpretation and replication of experiments. If you have any questions, please contact us: editorial@elifesciences.org.

Sample-size estimation

- You should state whether an appropriate sample size was computed when the study was being designed
- You should state the statistical method of sample size computation and any required assumptions
- If no explicit power analysis was used, you should describe how you decided what sample (replicate) size (number) to use

Please outline where this information can be found within the submission (e.g., page numbers or figure legends), or explain why this information doesn't apply to your submission:

There are no animal studies in this paper, so sample size estimation/power analysis does not apply.

Replicates

- You should report how often each experiment was performed
- You should include a definition of biological versus technical replication
- The data obtained should be provided and sufficient information should be provided to indicate the number of independent biological and/or technical replicates
- If you encountered any outliers, you should describe how these were handled
- Criteria for exclusion/inclusion of data should be clearly stated
- High-throughput sequence data should be uploaded before submission, with a private link for reviewers provided (these are available from both GEO and ArrayExpress)

Please outline where this information can be found within the submission (e.g., page numbers or figure legends), or explain why this information doesn't apply to your submission:

Independent biological replicates are defined by independent growth of worms. Technical replicates are defined as multiple analyses performed on one biological replicate, such as three independent measurements of RNA expression by qRT-PCR for RNA isolated on the same day. The biological replicates for each experiment are described in the main body of the text (for RNA-Seq), the figure legends (1, 3, 4, 5 and 6).

High-throughput sequencing data has been uploaded to GEO. The accession number and reviewer link are provided in the methods (Data Deposit section).

Statistical reporting

- Statistical analysis methods should be described and justified
- Raw data should be presented in figures whenever informative to do so (typically when N per group is less than 10)
- For each experiment, you should identify the statistical tests used, exact values of N, definitions of center, methods of multiple test correction, and dispersion and precision measures (e.g., mean, median, SD, SEM, confidence intervals; and, for the major substantive results, a measure of effect size (e.g., Pearson's r, Cohen's d)
- Report exact p-values wherever possible alongside the summary statistics and 95% confidence intervals. These should be reported for all key questions and not only when the p-value is less than 0.05.

Please outline where this information can be found within the submission (e.g., page numbers or figure legends), or explain why this information doesn't apply to your submission:

The raw data has been plotted as scatter plots for figures 1, 3, 4 and 6. The biological replicates and statistical test used for each figure is listed in the figure legend. Exact p-values are shown above the scatter plots in Figures 1, 3, 4 and 6.

(For large datasets, or papers with a very large number of statistical tests, you may upload a single table file with tests, Ns, etc., with reference to page numbers in the manuscript.)

Additional data files ("source data")

- We encourage you to upload relevant additional data files, such as numerical data that are represented as a graph in a figure, or as a summary table
- Where provided, these should be in the most useful format, and they can be uploaded as "Source data" files linked to a main figure or table
- Include model definition files including the full list of parameters used
- Include code used for data analysis (e.g., R, MatLab)
- Avoid stating that data files are "available upon request"

eLIFE
1st Floor
24 Hills Road
Cambridge CB2 1JP, UK
P 01223 855340
W elifesciences.org
T @elife

Please indicate the figures or tables for which source data files have been provided:

All editing sites identified by *SAILOR* and Sanger Sequencing are presented in Supplemental Document 1.

The differentially regulated genes that are summarized on page 10 are presented, with fold changes, in Supplemental Document 2.

The code for the software developed (*SAILOR*) to identify A-to-I editing sites is publically available (https://github.com/YeoLab/sailor) and the link is provided in the methods (*SAILOR* software section)

eLife Sciences Publications, Ltd is a limited liability non-profit non-stock corporation incorporated in the State of Delaware, USA, with company number 5030732, and is registered in the UK with company number FC030576 and branch number BR015634 at the address 1st Floor, 24 Hills Road, Cambridge CB2 1JP | August 2014
